# Implications of COVID-19 Innovations for Social Interaction: Provisional Insights From a Qualitative Study of Ghanaian Christian Leaders

**DOI:** 10.3389/fpsyg.2022.647979

**Published:** 2022-05-24

**Authors:** Glenn Adams, Annabella Osei-Tutu, Adjeiwa Akosua Affram, Lilian Phillips-Kumaga, Vivian Afi Abui Dzokoto

**Affiliations:** ^1^Department of Psychology, The University of Kansas, Lawrence, KS, United States; ^2^Post-Doctoral Fellowship-Programme, Goethe University Frankfurt, Frankfurt, Germany; ^3^Department of Psychology, University of Ghana, Accra, Ghana; ^4^Department of African American Studies, Virginia Commonwealth University, Richmond, VA, United States

**Keywords:** COVID-19, interpersonal contact, pandemic innovations, relationality, virtual interaction

## Abstract

Responses to the COVID-19 pandemic prompted people and institutions to turn to online virtual environments for a wide variety of social gatherings. In this perspectives article, we draw upon our previous work and interviews with Ghanaian Christian leaders to consider implications of this shift. Specifically, we propose that the shift from physical to virtual interactions mimics and amplifies the neoliberal individualist experience of abstraction from place associated with Eurocentric modernity. On the positive side, the shift from physical to virtual environments liberates people to selectively pursue the most fulfilling interactions, free from constraints of physical distance. On the negative side, the move from physical to virtual space necessitates a shift from material care and tangible engagement with the local community to the psychologization of care and pursuit of emotional intimacy in relations of one’s choosing—a dynamic that further marginalizes people who are already on the margins. The disruptions of the pandemic provide an opportunity to re-set social relations, to design ways of being that better promote sustainable collective well-being rather than fleeting personal fulfillment.

## Introduction

Observers have noted that the pandemic and response have accelerated the pace of developments that were already in motion before the pandemic (Roy, 2020). One of these developments concerns the technological mediation of social interaction. Although cultural innovations from writing to the telephone have increased the capacity of people to interact at a distance, responses to the pandemic prompted people and institutions to turn to online virtual environments for a wide variety of social gatherings: from work meetings to play dates, academic lectures to fitness classes, and religious services to nightclub outings. In this paper, we draw on theory and research in cultural psychology to consider possible consequences of this development for the experience of relationality.

The theoretical framework that informs our observations proposes that everyday life in Eurocentric global modernity promotes an experience of abstraction from context, freedom from constraint, and relational mobility—perceptions about opportunities and choice to form as well as terminate personal relationships (Yuki et al., 2007; Yuki and Schug, 2012)—associated with neoliberal individualist modes of being (Adams et al., 2019). At first glance, it might seem obvious that lockdown restrictions and confinement to social bubbles would limit mobility. This is especially true if one thinks of mobility in terms of movement across space or opportunities for physical encounters with other people. However, there is a somewhat paradoxical way in which the move to online virtual space increases relational mobility, in the sense of both opportunities for interaction and choice of interaction partners. Given that one must do social interactions or join events online, geographic distance poses few constraints. For example, the pandemic has likely enabled many readers in academic settings to attend webinars or lectures to which they only had access because restrictions on physical gatherings caused distant institutions to conduct these activities online. Similarly, a teenage child of one of the authors now spends more time in virtual social interaction with friends in Germany, Malaysia, and Canada than they do tangible social interaction with classmates from their secondary school who live around the corner. Simply put, interactions in the virtual world have qualities associated with cultural ecologies of neoliberal individualism and the corresponding experience of abstraction from place and high relational mobility (Adams et al., 2019). Our previous work has considered implications of such cultural ecologies for the experience of relationality.

One way to think about these consequences is to consider implications of mobility and abstraction from place for the construction and experience of interpersonal contact. An authoritative source defines the primary meaning of the English word *contact* as “union or junction of surfaces” (Merriam Webster, 2020) and it traces the etymological origins to Latin roots that have connotations of physical touch. Secondary meanings of *contact* refer to more contemporary connotations of *association*, *relationship*, *communication*, or *connection* between separate entities. It is plausible that these etymological shifts from primary to secondary meanings of contact corresponded to historical shifts as technological innovations enabled interpersonal interaction from a distance. For much of human history, interpersonal interaction required co-presence, with people necessarily restricted to interactions with others with whom they were within sight or shouting distance. Beginning with writing or other forms of communication through material symbols and then taking on a more interactive quality with the advent of telephones, technology made possible interpersonal interaction at a distance between people who were not co-present. These technologies enabled people to initiate contact or stay in figurative touch with people who were too distant for literal touch.

Associated with this shift from literal to figurative contact are consequences for the materiality of relationship (Esiaka and Adams, 2020). The abstraction of social interaction from place implies a shift in the activity of *sharing* such that it becomes less about joint participation in a common tangible experience and more about simultaneous interaction in parallel experiences linked through verbal exchange about the experience (see Mesquita, 2001, for a similar perspective with respect to cultural variation in affordances for emotion experience). Likewise, the abstraction of social interaction from place associated with ecologies of high relational mobility implies a shift in performance of care away from tangible forms of material assistance to verbally mediated emotional support (Adams and Plaut, 2003; Adams and Kurtiş, 2015; Esiaka and Adams, 2020).

## An Investigation of Christian Leaders in Ghana

An opportunity to investigate questions about the cultural-ecological foundations of social interaction arose in the context of an interview study of religious leaders that we were conducting in Ghana when the pandemic began. Health officials confirmed the first cases of COVID-19 in Ghana in March 2020. In response, the government of Ghana imposed a ban on all public gatherings, including mass gatherings for religious purposes, and instituted a partial lockdown in major cities (Ministry of Health, 2020). Given the centrality of religion and spirituality in everyday life for many African communities (Mbiti, 1969; Nwoye, 2015), we were curious about the steps that religious leaders took to carry out their mission during a lockdown period that encompassed some of the most sacred holidays on the Christian calendar (e.g., Easter). Because of the lockdown, we contacted potential participants through previous research contacts and referrals, and we conducted interviews through phone calls between May 15, 2020 and 29, 2020. We ended data collection when the president of Ghana announced a partial lifting of restrictions on religious gatherings (Communications Bureau, 2020).

Participants were 14 Christian leaders (11 men and three women; age range 34–60 years; *M* = 45.86, *SD* = 8.81) who had been in positions between 5 and 32 years (*M* = 16.54, *SD* = 8.35). They served Christian congregations that ranged in size from 50 to 600 people (*M* = 237.67; *SD* = 161.74) and were mostly based in urban communities (*n* = 11).^1^ We conducted interviews in a typical everyday mixture of English, the official language of Ghana, and Twi, a widely spoken Ghanaian language, depending on the preference of the participant. Among other prompts, we asked participants what alternative arrangements they made to provide services to their members, how they contacted people who were unable to access the new alternatives, and what form they thought religious life would take after the government lifted restrictions.

### Pandemic Innovations

As a response to the ban, most participants in our study (12 of 14) explicitly reported that they moved at least some activities online. These included conventional activities, such as posting prayer topics, but six participants reported that they livestreamed services for audiences who wished to view them synchronously (such as Facebook Live) or posted recordings of the services online (e.g., YouTube) for congregants to access at their convenience. The response of one man is particularly useful to illustrate the range of activities that different leaders reported:

We have been using the Facebook Live and some of the times too we do voice messages on our WhatsApp platforms. So, Sunday service we do it online and then Wednesday and Friday we do it online and then we also have special MoMo number (for electronic Mobile Money transfers) that we give to the members, encouraging them on our WhatsApp platforms to give.

The phrase “MoMo” refers to Mobile Money, a system for electronic funds transfers. In the absence of a physical service that would include opportunities for tangible donation (whether by passing around an offering basket or *via* more ostentatious displays of financial beneficence), this innovation provided a means for audience members to perform their duty of financial contribution to the church—a crucial activity for the survival of the organization about which the leaders we interviewed had particularly pressing concerns.

### Disadvantages of Virtual Interaction

It is perhaps noteworthy that the source of the preceding quote was the leader of an urban church in a relatively affluent neighborhood adjacent to the University of Ghana. The broad use of online affordances may be a function of a congregation with a relatively high proportion of well-educated members who have the required technology and expertise. Indeed, participants noted problems with access as an important constraint or disadvantage of the move to online virtual interaction. Although internet usage and smartphone technology are widespread in Ghana—estimates suggest that about 80 percent of the adult population in Ghana own a phone, and smartphone ownership is at 35 percent (Silver and Johnson, 2018)—there are disparities in access. People with financial constraints cannot afford either the hardware or data units for online virtual interaction. Beyond financial constraints, a male participant noted other constraints.

I realized that some of them have children… [who] have this online phone. So, I call them and I ask them that they have to connect their parents to the network… There are those who are also using the Android but they are not inclined to technology, so I have to do series of video tutorials given to them. Many have these challenges. Some too are complaining they have it, but where they are located, the network does not allow them to hear the flow.

Even if people have the necessary equipment, network coverage is typically less widespread or dependable in rural areas than in urban areas. Moreover, even in settings with required equipment and network capabilities, expertise required for effective virtual engagement is typically lower among elders than youth. In these cases, one solution is a hybrid situation in which small groups of members, often from the same household or family, meet physically together in separate pods to join virtual services from a distance mediated by internet technology.

Besides inequality of access, another disadvantage of the move from physical to virtual meeting space concerns the depth and quality of social interactions. Some of the religious leaders in our study expressed dissatisfaction with the quality of online services. For example, a male preacher noted that,

When you are preaching without congregation, it’s not the same [when] you are preaching to the machine. The congregation, when you are preaching directly, it has some special impact because you are hearing the people. … That is the challenges (*sic*) you have.

As instructors of online classes can no doubt relate, this participant suggested that the experience of preaching to a virtual audience was less satisfying and perhaps less effective because it lacked the energy and feedback associated with the tangible audience participation. Emoticons announcing that an audience member finds a speaker’s joke humorous or symbols of hands clapping are a poor substitute for hearing tangible laughter or audience applause.

Our interviews with religious leaders did not provide an audience perspective on the experience of virtual religious gatherings, but participant observation and informal conversation suggest that many audience members again find them a poor substitute for the experience of physical gatherings.

There are few things more powerful than being in the presence of a Black gospel choir, its lead singer clapping and moving in rhythm testifying to the power of God. There are moments when the choirs and the preachers who follow can lift an entire congregation and transport it. They can fill the despairing with hope and the fearful with the courage to demand justice (McCauley, 2020).

This energy and tangible materiality of physical gatherings is difficult to reproduce in virtual gatherings, where “instead of choirs, we mumble along trying to harmonize with a virtual worship leader” (McCauley, 2020).

### Advantages of Virtual Interaction

Despite disadvantages, participants also noted benefits of the move to virtual interaction, associated with freedom from place-based constraints that are directly relevant to the theoretical considerations that animated our investigation. One such benefit was an increase in the volume of participation, as a male participant described.

What we have also realized is that, the online, you have a larger audience. Because in our church we are about, let us say 400. When you put the message on Facebook and other social media, you will find out that you have more than [that]. Sometimes by the close of the week we have about 1,000 [attendees].

Pandemic innovations meant that potential audience members were no longer constrained to attend services at a particular place and time, but instead were free to access services and other activities at a time and from a source of their choosing—even activities based in locales thousands of kilometers away. Participants noted that the convenience of virtual interaction resolved other barriers to attendance.

When it comes to the advantages too … those who are participating are very happy because they sit at the comfort of their homes, they are not late just like they come to church, they have to pick cars. This time they are able to participate early and they could hear everybody clearly if their network is okay.

Whereas physical interaction in standard services requires that people rise early to groom themselves and to negotiate uncertainties of traffic or public transportation, the relative convenience of virtual participation removed these barriers to attendance.

## Discussion

After the Ghanaian government eased restrictions on public gatherings, churches, and other places of worship resumed joint religious activities in physical space (albeit with strict protocols for disinfectant cleaning and social distancing). Yet, churches continued to conduct some activities in virtual space not only because of reduced capacity for physical attendance, but also because some members desire the option of virtual gatherings. More generally, the increased online presence spurred by the pandemic shows little sign of abating; some features, like affordances for virtual financial contributions, are likely to be permanent innovations.

Even so, our purpose here is not to argue that pandemic innovations have fundamentally and irreversibly altered religious or social interaction practices. Instead, our purpose is to consider whether and how pandemic innovations amplify or set in high relief broader cultural changes that were already in motion. In particular, we propose that innovations for social interaction during the pandemic offer a window into broader changes in social relations associated with the neoliberal individualism of everyday life in the Eurocentric modern order. The experience of relational mobility and abstraction of social relations from context associated with both virtual space and cultural ecologies of neoliberal individualism means that people are free to connect with a greater number and wider range of interaction partners. The relatively frictionless character of these cultural ecologies means that people can shop around to find the most entertaining or most fulfilling religious or social interactions with a minimum of commitment, knowing that they can easily exit interactions or religious communities and search for new ones if the old ones are longer fulfilling or have become too burdensome.

Although this ability to “shop around” has potential benefits for individual optimization of satisfaction and fulfillment, it also has important costs. After all, the importance of choice as a determinant of social connections comes with a corresponding imperative to be chosen (Plaut et al., 2009). Social interactions that are insufficiently attractive or excessively burdensome—communities who have too little to offer or require too much cost and care—are at risk of abandonment as people exercise their options to find a better deal (Livingston, 2019). This dynamic amplifies a shift away from the materiality of care (Coe, 2011) or tangible social interactions with family and local community toward the psychologization of care and emphasis on emotional intimacy in relations of one’s choosing (Salter and Adams, 2012; Esiaka et al., 2020; Osei-Tutu et al., 2022). Whether for religious communities or individual people, the result of this dynamic is to exacerbate exclusion and inequality, to further marginalize those who are already on the margins.

Some authors have discussed the idea that “the pandemic is a portal” (Roy, 2020; see also Mbembe, 2020). This is not so much about prediction of the future as it is a call to reset: to recognize, to reflect on, and then to change destructive cultural-psychological habits. The pandemic-fueled migration of social life from physical to virtual interactions illuminated the benefits and costs of the neoliberal individualist emphasis on psychologization of care and emotional support, at the expense of tangible care and material support that characterizes relationality in the modern global order. With this knowledge in hand, we have an opportunity to design forms of social interaction and relationality that strike a more appropriate balance between opportunities for personal fulfillment and practices that ensure sustainable collective well-being.

## Data Availability Statement

The raw data supporting the conclusions of this article will be made available by the authors, without undue reservation.

## Ethics Statement

The studies involving human participants were reviewed and approved by Ethics Committee for the Humanities, University of Ghana. Written informed consent for participation was not required for this study in accordance with the national legislation and the institutional requirements.

## Author Contributions

AO-T contributed to the study design, data collection, data analyses and interpretation, and writing of the manuscript. GA contributed to the study design, data analyses and interpretation, and writing of the manuscript. AA contributed to the study design and data collection and analyses. LP-K contributed to the writing of the manuscript. VD contributed to the study design and data interpretation. All authors contributed to the article and approved the submitted version.

## Funding

This study was made possible through the support of a grant from the Volkswagen Foundation Knowledge for Tomorrow—Cooperative Research Projects in Sub-Saharan Africa Program (grant number 94667).

## Conflict of Interest

The authors declare that the research was conducted in the absence of any commercial or financial relationships that could be construed as a potential conflict of interest.

## Publisher’s Note

All claims expressed in this article are solely those of the authors and do not necessarily represent those of their affiliated organizations, or those of the publisher, the editors and the reviewers. Any product that may be evaluated in this article, or claim that may be made by its manufacturer, is not guaranteed or endorsed by the publisher.

## Footnotes

^1^We subsequently interviewed several Islamic clerics (n = 19) about their use of communication technology during the COVID lockdown. Consistent with observations from other settings (e.g., Akmaliah and Burhani, 2021; Kühle and Larsen, 2021), the Islamic clerics whom we interviewed reported relatively scant adoption of communication technology, mostly limited to phone discussions and online posting of content rather than synchronous virtual gatherings of videoconferences.

## References

[ref1] Adams G. Estrada-Villalta S. Sullivan D. Markus H. R. (2019). The psychology of neoliberalism and the neoliberalism of psychology. J. Soc. Issues 75, 189–216. doi: 10.1111/josi.12305, PMID: 34931937

[ref2] Adams G. Kurtiş T. (2015). Friendship and gender in cultural-psychological perspective: implications for research, practice, and consultation. Int. Perspect. Psychol. Res. Pract. Consult. 4, 182–194. doi: 10.1037/ipp0000036

[ref3] Adams G. Plaut V. C. (2003). The cultural grounding of personal relationship: friendship in north American and west African worlds. Pers. Relat. 10, 333–347. doi: 10.1111/1475-6811.00053, PMID: 15982115

[ref4] Akmaliah W. Burhani A. N. (2021). Digital Islam in Indonesia: The shift of ritual and religiosity during COVID-19. ISEAS Perspective 12. Available at: https://www.iseas.edu.sg/articles-commentaries/iseas-perspective/2021-107-digital-islam-in-indonesia-the-shift-of-ritual-and-religiosity-during-covid-19-by-wahyudi-akmaliah-and-ahmad-najib-burhani/ (Accessed May 10, 2022).

[ref5] Coe C. (2011). What is love? The materiality of care in Ghanaian transnational families. Int. Migr. 49, 7–24. doi: 10.1111/j.1468-2435.2011.00704.x, PMID: 22180882

[ref6] Communications Bureau (2020). Update No.10: Measures taken to combat spread of coronavirus. Available at: http://presidency.gov.gh/index.php/briefing-room/speeches/1597-update-no-10-measures-taken-to-combat-spread-of-coronavirus (Accessed May 10, 2022).

[ref7] Esiaka D. Adams G. (2020). Epistemic violence in research on elder care. Psychol. Dev. Soc. 32, 176–200. doi: 10.1177/0971333620936948

[ref8] Esiaka D. Adams G. Osei-Tutu A. (2020). Dilemma tales as African knowledge practice: an example from research on obligations of support. Front. Psychol. 11:546330. doi: 10.3389/fpsyg.2020.546330, PMID: 33132955 PMC7561668

[ref9] Kühle L. Larsen T. L. (2021). ‘Forced’ online religion: religious minority and majority communities’ media usage during the COVID-19 lockdown. Religion 12:496. doi: 10.3390/rel12070496

[ref10] Livingston J. (2019). Self-Devouring Growth: A Planetary Parable as Told From Southern Africa. Durham, NC, USA: Duke University Press.

[ref11] Mbembe A. (2020). A universal right to breathe. Crit. Inq. 47, S58–S62. doi: 10.1086/711437

[ref12] Mbiti J. S. (1969). African Religions and Philosophy. London, UK: Heinemann.

[ref13] McCauley E. (2020). Why You Can’t Meet God Over Zoom: Virtual Services are Inadequate—But I Keep Going. The New York Times, Available at: https://www.nytimes.com/2020/12/24/opinion/zoom-church-christmas-covid-loss.html?campaign_id=9andemc=edit_nn_20201224andinstance_id=25372andnl=the-morningandregi_id=21604506andsegment_id=47712andte=1anduser_id=d68c9ef3aa2d914a3613be569cfebb34 (Accessed May 10, 2022).

[ref14] Merriam Webster (2020). Contact. Available at: https://www.merriam-webster.com/dictionary/contact (Accessed May 10, 2022).

[ref15] Mesquita B. (2001). Emotions in collectivist and individualist contexts. J. Pers. Soc. Psychol. 80, 68–74. doi: 10.1037/0022-3514.80.1.68, PMID: 11195892

[ref16] Ministry of Health (2020). President Akufo-Addo addresses nation on measures taken by gov’t to combat the coronavirus pandemic. Available at: https://www.moh.gov.gh/president-akufo-addo-addresses-nation-on-measures-taken-by-govt-to-combat-the-coronavirus-pandemic/ (Accessed May 10, 2022).

[ref17] Nwoye A. (2015). What is African psychology the psychology of? Theory Psychol. 25, 96–116. doi: 10.1177/0959354314565116, PMID: 35511542

[ref18] Osei-Tutu A. Adams G. Esiaka D. Dzokoto V. A. Affram A. A. (2022). The modernity/coloniality of love: individualist selfways and charismatic Christianity in Ghanaian worlds. J. Soc. Issues 78, 183–208. doi: 10.1111/josi.12432

[ref19] Plaut V. C. Adams G. Anderson S. L. (2009). Does beauty buy well-being? It depends on where you're from. Pers. Relat. 16, 619–630. doi: 10.1111/j.1475-6811.2009.01242.x

[ref20] Roy A. (2020). The pandemic is a portal. Financial Times, 3.

[ref1800] Salter P. S. Adams G. (2012). Mother or wife? An African dilemma tale and the psychological dynamics of sociocultural change. Soc. Psychol. 43, 232–242. doi: 10.1027/1864-9335/a000124

[ref21] Silver L. Johnson C. (2018). Internet connectivity seen as having positive impact on life in Sub-Saharan Africa. Available at: https://www.pewresearch.org/global/2018/10/09/internet-connectivity-seen-as-having-positive-impact-on-life-in-sub-saharan-africa/#table (Accessed May 10, 2022).

[ref22] Yuki M. Schug J. (2012). “Relational mobility: a socioecological approach to personal relationships,” in Relationship Science: Integrating Evolutionary, Neuroscience, and Sociocultural Approaches. eds. Gillath O. Adams G. Kunkel A. (Washington, DC, USA: American Psychological Association), 137–151.

[ref23] Yuki M. Schug J. Horikawa H. Takemura K. Sato K. Yokota K.. (2007). Development of a Scale to Measure Perceptions of Relational Mobility in Society. CERSS Working Paper 75, Center for Experimental Research in Social Sciences, Hokkaido University.

